# Protective Effects of *Polygonatum sibiricum* Polysaccharides Against Type 2 Diabetic Mice Induced by High-Fat Diet and Low-Dose Streptozotocin

**DOI:** 10.3390/toxics13040255

**Published:** 2025-03-28

**Authors:** Qingxiangzi Li, Jufen Cheng, Yangyang Sun, Liang He, Rui Li

**Affiliations:** 1 Laboratory Animal Center, Zhejiang Academy of Agricultural Sciences, Hangzhou 310021, China; liqxzi@163.com; 2 Institute of Animal Husbandry and Veterinary Science, Zhejiang Academy of Agricultural Sciences, Hangzhou 310021, China; 3 Xianghu Laboratory, Hangzhou 311231, China; 4 Institute of Agro-Product Safety and Nutrition, Zhejiang Academy of Agricultural Sciences, Hangzhou 310021, China

**Keywords:** *Polygonatum sibiricum* polysaccharide, diabetes, glycolipid metabolism, gut microbiota

## Abstract

Polysaccharides possessing hypoglycemic effects have shown promising results in treating diabetes. *Polygonatum sibiricum* polysaccharide (PSP) is one of the most active ingredients in the Chinese medicine *P. sibiricum* Redoute with many biological activities. However, its efficacy in alleviating type 2 diabetes mellitus (T2DM) remains unexplored. Our aim is to evaluate the protective effect of PSP against T2DM by measuring body weight and serum biochemical indicators, examining the histopathological images of pancreatic and liver tissues, detecting fecal short-chain fatty acid (SCFA) content, and analyzing the intestinal flora diversity and the microbiota structure in T2DM mice. The findings indicated that PSP treatment in T2DM mice could obviously decrease the fasting blood glucose and fasting insulin levels, ameliorate glucose tolerance, insulin resistance, lipid, and inflammatory factor levels, attenuate pancreatic and liver damage, and increase the fecal SCFA content. In addition, PSP could modulate the composition of gut microbiota in T2DM mice, resulting in the relative abundance of *Firmicutes* decreasing and that of *Bacteroidetes* increasing, along with the abundance of beneficial flora significantly increasing, especially SCFA-producing bacteria. The findings indicate that PSP administration protected against diabetes by controlling disordered glucolipid metabolism and modulating the gut microbiota, which provides a valuable strategy for the utilization of PSP to treat T2DM.

## 1. Introduction

As a serious metabolic disorder, diabetes mellitus is commonly characterized by continuous hyperglycemia, resulting in atherosclerosis, renal failure, neuropathy, retinopathy, etc. [1]. Diabetes mellitus affected 536.6 million people in 2021, and its prevalence continues to increase worldwide [2]. The most predominant form is type 2 diabetes mellitus (T2DM), which affects most diabetic patients [3]. The T2DM pathogenesis is mainly insulin resistance (IR) and decreased insulin secretion caused by structural changes or a functional impairment of pancreatic β-cells [4]. T2DM is closely linked with a characteristic pattern of dyslipidemia, hypertension, nephrology, intestinal flora disorders, etc. [5]. Currently, oral hypoglycemic medicines and injections of exogenous insulin are generally used for diabetes mellitus treatment. However, the use of antidiabetic drugs is correlated mostly with tolerance problems and toxic side effects after long-term use [6]. Therefore, the recognition of effective, safe, and healthy dietary regimens and medical therapies for T2DM is a public health priority.

Traditional Chinese medicine (TCM), with over two thousand years of development, has been more widely used in the last several decades worldwide [7]. In the last few years, many studies on TCM have been performed to prevent and manage diabetes, and the effectiveness and benefits of TCM have been confirmed [8]. Recently, some studies have shown that bioactive dietary polysaccharides, such as tea polysaccharides, *Ganoderma lucidum* polysaccharides, and *Cyclocarya paliurus* polysaccharides, are effective at alleviating hyperglycemia, hyperlipidemia, and insulin resistance in T2DM model animals, with fewer adverse effects [9,10,11]. Thus, polysaccharide intervention is considered an alternative for treating diabetes mellitus. *Polygonatum sibiricum* Redoute, known as “Huangjing” in TCM, is a perennial herb with medicinal and edible homology [12]. As one of the important active compounds of this plant, *P. sibiricum* polysaccharide (PSP) has various pharmacological actions, including antiaging, antioxidant, antitumor, anti-inflammatory, and immunomodulatory properties [13,14]. Moreover, research conducted by Zeng et al. revealed that PSP protected against nonalcoholic fatty hepatopathy and obesity in rats that consumed fatty foods [15]. Wang et al. reported that PSP potentially alleviated retinal injury in rats with diabetes [16]. Nevertheless, knowledge of the effects of PSP on diabetic mice is limited, and the mechanism of action of PSP in treating T2DM remains unclear.

Intestinal microorganisms comprise trillions of bacteria, helping maintain a balance of energy and good health of the body [17]. Recently, some researchers believed that gut microbiota dysbiosis affected diabetic progression [18]. Increasing the abundance of conditionally pathogenic bacteria and decreasing the abundance of beneficial bacteria altered the gut microbiota structure in diabetic mice [19]. The disruption of the gut microbiota impairs gut barrier function and increases the level of the endotoxin lipopolysaccharide in the bloodstream, resulting in the exacerbation of insulin resistance and systemic inflammation to some extent [20]. Notably, a dietary polysaccharide intervention could ameliorate diabetes mellitus by affecting the composition of gut microbiota, elevating the short-chain fatty acid (SCFA) levels derived from bacterial metabolism, enhancing the intestinal barrier integrity, maintaining intestinal immune homeostasis, promoting the secretion of gut hormones, and altering bile acid secretion [21]. In addition, the monomeric polysaccharide from *P. sibiricum* was found to reshape the gut microbiota by increasing the abundance of *Akkermansia muciniphila* and decreasing *Helicobacter* in mice with Alzheimer’s disease [22]. Nevertheless, to our knowledge, studies on the PSP-mediated modulation of the gut microbiota are limited, especially in individuals with T2DM.

Our objective is to investigate the protective effects of PSP on a T2DM mouse model induced by feeding a high-fat diet (HFD) combined with a streptozotocin (STZ) injection, and the potential mechanisms involved in regulating glycolipid metabolism and the gut microbiota were explored. The findings of this study give novel insights into PSP development as a therapeutic drug and functional food for diabetes mellitus.

## 2. Materials and Methods

### 2.1. Materials

PSP (purity ≥ 98%) is a commercial product purchased from Shanxi Ci Yuan Biotechnology Corporation (Xianyang, China) with no competing financial interests. Metformin (purity ≥ 97%) and STZ (purity ≥ 98%) were obtained from Sigma–Aldrich Corporation (St. Louis, MO, USA). Jiangsu Xietong Pharmaceutical Bio-engineering Corporation (Nanjing, China) provided standard feed and a high-fat diet. ELISA kits for detecting fasting insulin (FINS), mouse tumor necrosis factor-α (TNF-α), mouse interleukin-6 (IL-6), and C-reactive protein (CRP) levels were obtained from ZCIBIO Technology Corporation (Shanghai, China). The glucometer was procured from Roche Diagnostics GmbH (Mannheim, Germany). Moreover, the Service Biotechnology Corporation (Wuhan, China) supplied the test kits for serum high-density lipoprotein cholesterol (HDL-C), low-density lipoprotein cholesterol (LDL-C), total cholesterol (TC), and total triglyceride (TG) measurements.

### 2.2. Animal Experimental Design

**Figure 1:**
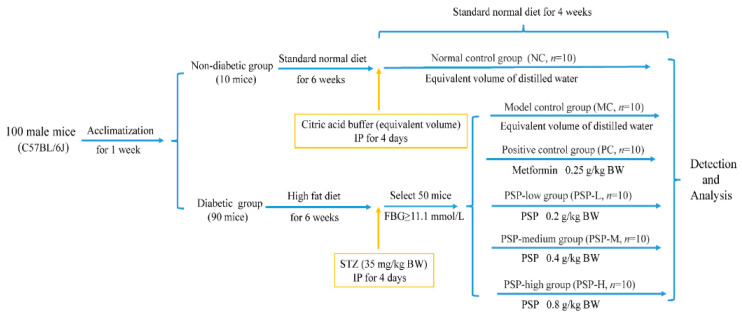
Design of the animal experiment.

One hundred male C57BL/6J mice (4–5 weeks) were provided by the Zhejiang Vital River Laboratory Animal Technology Corporation (No. SCXK (Zhe) 2020-0002, Jiaxing, China). Mice were housed in a standard animal facility with a light/dark cycle (12 h) at about 25 °C and 60% humidity, and water and standard feed were available ad libitum. HFD can induce insulin resistance, and STZ specifically targets and destroys the insulin-producing β-cells of the pancreas, resulting in markedly impaired insulin secretion and hyperglycemia. Thus, HFD and STZ were used for establishing the animal model with T2DM. After 7 days of acclimatization, the normal control (NC) group consisting of 10 randomly selected mice was fed a standard diet. Other mice were used to establish the diabetic model and were fed a high-fat diet for 6 weeks (composition: 38% maintenance base, 28% lard, 5.6% sucrose, 10.8% whole milk powder, 11.5% casein, 1.9% microcrystalline cellulose, 2% laboratory animal premix, 1.8% calcium phosphate, and 0.4% rock flour). STZ was freshly prepared using citric acid buffer (pH 4.5, 0.1 mol/L). The model group was administered an intraperitoneal injection (IP) of STZ (35 mg/kg of body weight (BW)) for 4 consecutive days, whereas the NC group was administered an equal amount of citric acid buffer. Ten days later, fasting blood glucose (FBG) levels in blood from the tail vein were measured. Then, 50 animals with FBG over 11.1 mmol/L were selected and randomized into 5 groups (gavage, *n* = 10): the T2DM model control (MC; distilled water), positive control (PC; 0.25 g/kg BW metformin), PSP-low (PSP-L; 0.2 g/kg of BW PSP), PSP-medium (PSP-M; 0.4 g/kg of BW PSP) and PSP-high (PSP-H; 0.8 g/kg of BW PSP) groups. PSP was diluted by distilled water at the required concentration of 20, 40, and 80 mg/mL, and then stored in a refrigerator at 4 °C. PSP was reconstituted in 40 °C water-bath heating for 5 min and then shocked before oral gavage. Metformin was dissolved in the sterile distilled water in the final concentration of 25 mg/mL before oral gavage. Each group of mice was treated once daily for 4 weeks, and the NC group was administered distilled water. In addition, food and water intake in all groups was monitored weekly during drug administration. Five mice were kept in one clean cage. Food intake was measured by weighing the loss of the feed for 3 consecutive days to calculate the daily average values. Water intake was measured by weighing the loss in water volume using 100 mL graduated water bottles for 3 consecutive days to calculate the daily average values. The animal experimental design is presented in Figure 1.

At the experimental endpoint, mice were placed in metabolic cages for collecting fecal samples. One metabolic cage was used for each individual mouse, and a total of 3–5 fecal pellets from each mouse were collected into sterilized centrifuge tubes and then immediately placed in liquid nitrogen for subsequent analysis. Before being killed, the mice in all the groups were fasted overnight (12 h). Blood samples were collected from the retro-orbitary sinuses of the mice with isoflurane anesthesia. The collected blood was allowed to stand for 2 h at room temperature, and the serum was separated by centrifugation at 3000× *g* for 10 min. Mice were bled to death after deep anesthesia with isoflurane. After being killed, liver and kidney tissue samples were collected, washed with saline, and weighed to calculate organ indices. The liver and kidney indices were, respectively, calculated as the ratio of the liver and kidney weight to the body weight. Moreover, the 4% paraformaldehyde solution was used to store the pancreas and liver for analyzing the histopathological change.

### 2.3. BW, FBG Measurement, and Oral Glucose Tolerance Test (OGTT)

After acclimatization, the mice were weighed weekly. Before modeling, the initial FBG levels of the mice were measured, and the FBG levels of the diabetic mice were tested again after STZ induction. Changes in the mice’s FBG levels were subsequently recorded weekly during the drug intervention. The OGTTs were performed 2 days prior to the study endpoint as follows: After fasting overnight (12 h), glucose (gavage, 2 g/kg BW) was given to mice in all the groups, and then the blood samples were obtained through the tail vein at different time points (0, 30, 60, 90, and 120 min) to detect blood glucose values.

### 2.4. Measurement of Biochemical Indicators and Histopathological Analysis

Serum LDL-C, HDL-C, TG, and TC levels were measured by the automatic biochemical apparatus (Chemray 800, Rayto Life and Analytical Sciences Corporation, Shenzhen, China). The FINS, C-reactive protein, IL-6, and TNF-α levels were measured by the enzyme-linked immunosorbent assay kits. The mouse homeostasis model assessment of the insulin resistance index (HOMA-IR) was calculated as follows: HOMA-IR = FINS (mIU/L) × FBG (mmol/L)/22.5.

The 4% paraformaldehyde was used to fix pancreatic and liver tissues for 48 h, followed by trimming, dehydrating, and embedding in paraffin. Then, the 5 μm thick slices were cut and transferred into xylene to remove all paraffin. After dewaxing, the section was passed sequentially through jars of absolute alcohol until completely desiccated. The slides were washed briefly for 5 min, stained in hematoxylin for 15 min, followed by water washing for 8 min. Then, the slides were differentiated in 0.5% hydrochloric acid–alcohol solution for 10 s, washed by water for 20 min, stained in eosin–alcohol solution for 5 min, washed by water for 5 s, dehydrated by absolute alcohol for about 10 s, and the section was cleared with xylene. Finally, the slides were sealed and examined with a microscope (Olympus BX 50, Tokyo, Japan).

### 2.5. Fecal SCFA Analysis

One fecal sample (100 mg) was thawed and suspended in 1 mL of water, mixed with 0.2 mL of crotonic acid–metaphosphoric acid solution (646.4 mg of crotonic acid and 25.0 g of metaphosphoric acid dissolved in 100 mL of water), and then acidified at −40 °C for 24 h. The suspension was separated by centrifugation at 13,000× *g* for 3 min at 4 °C after thawing, filtered by the filtration membrane (0.22 μm), then injected into a gas chromatograph (Shimadzu, Kyoto, Japan) with a DB-FFAP column (Agilent Technologies, Santa Clara, CA, USA) for the determination of SCFAs. The internal standard was crotonic acid. The operating conditions were as follows: 250 °C of the inlet and the FID detector temperatures; 20 mL/min of the carrier gas flow rate with a 10:1 split ratio. In addition, the column temperature began at 80 °C and gradually increased to 190 °C (10 °C/min) with a hold for 0.5 min and then increased to 240 °C (40 °C/min) with a hold for 4 min.

### 2.6. Gut Microbiota Analysis

At the experimental endpoint, feces were randomly collected from 6 mice per group for 16S rRNA gene sequencing (Majorbio Bio-Pharm Technology Corporation, Shanghai, China). DNA from fecal samples was extracted by a DNA kit (Omega Bio-tek, Norcross, GA, USA). Bacterial 16S rRNA containing V3-V4 variable regions was subsequently amplified with specific primers 341F (5′-CCTAYGGGRBGCASCAG-3′) and 806R (5′-GGACTACNNGGGGTATCTAAT-3′). Purified PCR amplicons were sequenced on an Illumina NovaSeq PE250 platform (Illumina, San Diego, CA, USA). The NCBI SRA database downloaded the raw data (accession number: PRJNA1171983). Afterward, the original sequences were filtered using Fastp software (Version 0.19.6) and merged with Flash software (Version 1.2.11). The high-qualified data were obtained through denoising using the QIIME2 software (Version 2020.2) to generate amplicon sequence variants (ASVs) representing sequences and abundance information. Based on the ASV information, a bioinformatic analysis was carried out on the Majorbio Cloud platform (https://cloud.majorbio.com).

### 2.7. Statistical Analysis

Data are shown as the means ± standard deviations (SDs). Statistical analysis and graphical presentation were performed on SPSS 22.0 and GraphPad Prism 8.0, respectively. The Shapiro–Wilk test was used to determine whether the data came from a normal distribution. For normally distributed data, over time, the data of BW, FBG, and OGTT were analyzed by two-way ANOVA, and the data of food and water intake, liver index and kidney index, FINS, HOMA-IR, TG, TC, HDL-C, LDL-C, IL-6, TNF-α, CRP levels, and fecal SCFA contents were analyzed by one-way ANOVA coupled with the Duncan post hoc test to determine significant differences between multiple groups. For non-normally distributed data (Chao index and Shannon index, relative abundance of the gut microbiota at the phylum level and the genus level, and ratio of *Firmicutes* to *Bacteroidetes*), the Kruskal–Wallis test was used for analyzing differences between multiple groups.

## 3. Results

### 3.1. PSP Effects on the Mouse BW, Food and Water Intake, and Organ Index

**Figure 2:**
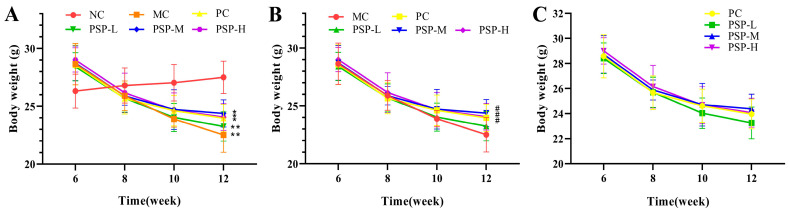
PSP effects on BW in T2DM mice. (**A**) * *p* < 0.05 or ** *p* < 0.01 indicates significant differences between NC and MC, PC, PSP-L, PSP-M, and PSP-H; (**B**) ^#^ *p* < 0.05 indicates significant differences between MC and PC, PSP-L, PSP-M, and PSP-H; (**C**) there is no significant difference in BW between the PC, PSP-L, PSP-M, and PSP-H groups.

**Figure 3:**
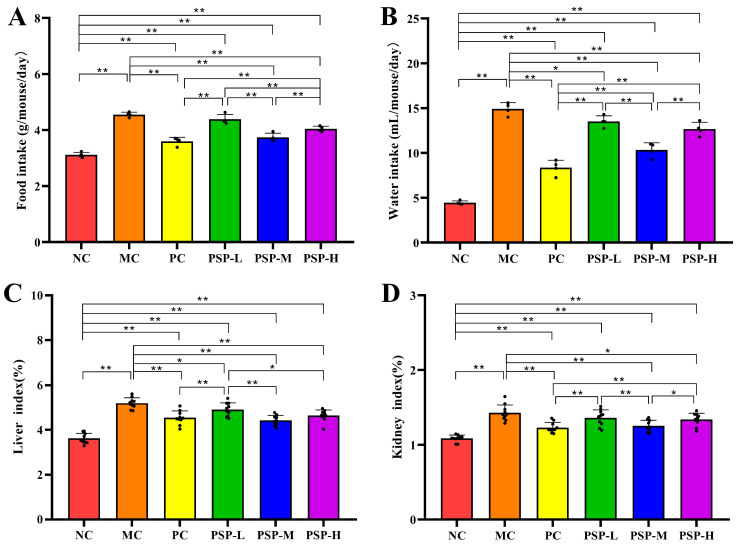
PSP effects on (**A**) food intake, (**B**) water intake, (**C**) the liver index, and (**D**) the kidney index. Note: * *p* < 0.05 or ** *p* < 0.01 indicates significant differences between groups, black dot (·) represents a data point.

The mice in the NC group presented a good mental status during the experiment. The mouse BW was not significantly different among these six groups at the beginning of the study (average of 21.87 g). The STZ-induced mice presented a decreased BW and increased food and water intake, which was consistent with typical diabetic symptoms [23]. Compared with the pre-dose weight (week 8), the average weight loss after 4 weeks of treatment reached 12.8%, 6.4%, 9.2%, 5.8%, and 7.7% in MC, PC, PSP-L, PSP-M, and PSP-H groups, respectively. As shown in Figure 2, the BW of the MC group was different from that of the NC (*p* < 0.01), and the BW of the PSP-M, H, and PC groups was different from that of the MC (*p* < 0.05). However, no marked differences in mice BWs were detected between the PC group and the three groups treated with different doses of PSP (*p* > 0.05). These findings indicated that PSP intervention mitigated weight loss in T2DM mice. Figure 3A,B show that the average food intake and water intake in the T2DM mice were greater than those in the healthy mice (*p* < 0.01), and after drug treatment, those values in the PC, PSP-M, and PSP-H groups decreased compared with those in the MC group (*p* < 0.01). In addition, as presented in Figure 3C,D, the liver and kidney indices of diabetic mice appeared to be usually greater than those of healthy mice, while PSP and metformin treatment suppressed this trend, and the organ index values in the three groups (PC, PSP-M, and PSP-H) decreased compared to the MC group at the experimental endpoint (*p* < 0.05).

### 3.2. PSP Effects on the FBG Levels and OGTT Results of Mice

**Figure 4:**
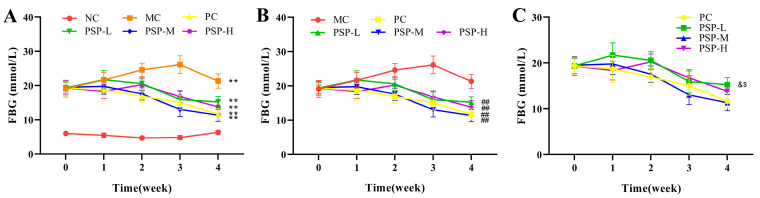
PSP effects on FBG in T2DM mice after administration. (**A**) ** *p* < 0.01 indicates significant differences between NC and MC, PC, PSP-L, PSP-M, and PSP-H; (**B**) ^##^ *p* < 0.01 indicates significant differences between MC and PC, PSP-L, PSP-M, and PSP-H; (**C**) ^&^ *p* < 0.05 indicates significant differences between PC and PSP-L, PSP-M, and PSP-H; ^$^ *p* < 0.05 indicates significant differences between PSP-M and PSP-L and PSP-H.

In terms of the FBG levels throughout the experiment, the initial FBG levels had no significant difference among all the groups. After STZ induction, the average FBG levels of mice in the five T2DM groups exceeded 11.1 mmol/L, showing that the hyperglycemia model was successfully established. As shown in Figure 4, one week after administration, only the diabetic mice in the PC and PSP-H groups presented slight decreases in mean FBG levels. After 3 weeks of the intervention, the mean FBG levels of the mice in all the drug-treated groups decreased relative to those at week 2, whereas the FBG level was still elevated in the MC group, indicating that the pancreatic β-cells were continually destroyed by STZ in T2DM mice. The FBG levels in the PC and the three PSP groups (PSP-L, PSP-M, and PSP-H) were obviously lower than that of the MC group (*p* < 0.01). Moreover, marked differences in the FBG level were found between the PC and PSP-L groups (*p* < 0.05), whereas no marked differences were found between the PC and PSP-M and H groups (*p* > 0.05). These results suggest that PSP had a good hypoglycemic effect, and the best hypoglycemic activity was observed in the PSP-M and PSP-H groups.

**Figure 5:**
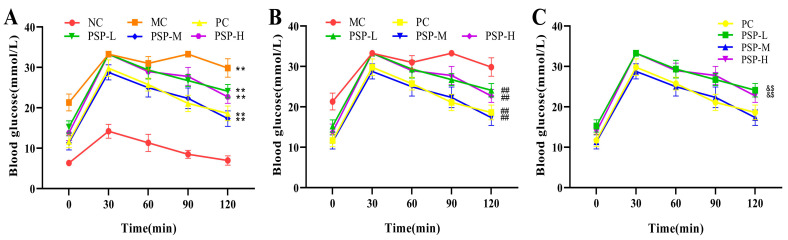
PSP effects on OGTT in T2DM mice. (**A**) ** *p* < 0.01 indicates significant differences between NC and MC, PC, PSP-L, PSP-M, and PSP-H; (**B**) ^##^ *p* < 0.01 indicates significant differences between MC and PC, PSP-L, PSP-M, and PSP-H; (**C**) ^&^ *p* < 0.05 indicates significant differences between PC and PSP-L, PSP-M, and PSP-H; ^$^ *p* < 0.05 indicates significant differences between PSP-M and PSP-L and PSP-H.

After oral glucose administration, the average blood glucose levels in all the mouse groups increased to the highest value at 30 min (Figure 5). Two hours after administration, the blood glucose levels in the NC group were in the normal range, and the mean blood glucose levels in the PSP- and metformin-treated groups decreased compared to the MC group (*p* < 0.01). The lowest blood glucose level (17.34 ± 1.92 mmol/L) was observed in the PSP-M group at 120 min. There were no marked differences between the PC and the PSP-M and PSP-H groups (*p* > 0.05). Our results suggest that PSP improved the T2DM mice’s glucose tolerance.

### 3.3. PSP Effects on FINS, HOMA-IR, and Serum Lipid Levels

**Figure 6:**
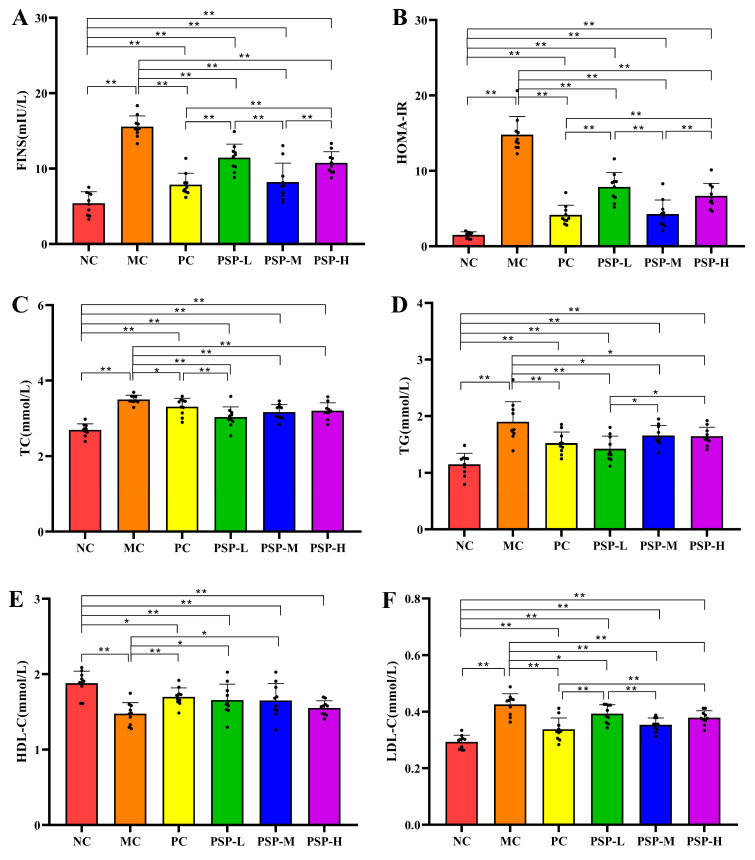
PSP effects on (**A**) FINS, (**B**) HOMA-IR, (**C**) TC, (**D**) TG, (**E**) HDL-C, and (**F**) LDL-C levels. Note: * *p* < 0.05 or ** *p* < 0.01 indicates significant differences between groups, black dot (·) represents a data point.

Figure 6A,B show that the FINS level and HOMA-IR in the MC group were higher than those of the NC group (*p* < 0.01). These levels (FINS and HOMA-IR) in all the drug-treated groups were lower than those of the MC group at the experimental endpoint (*p* < 0.01). Among them, metformin and 0.4 g/kg of BW PSP interventions had the best protective effects, with FINS levels of 7.88 ± 1.51 mIU/L and 8.24 ± 2.50 mIU/L and HOMA-IR values of 4.16 ± 1.29, and 4.28 ± 1.87 in these two groups, respectively. Additionally, as shown in Figure 6C–F, mice treated with drugs had different degrees of decreased LDL-C, TG, and TC levels and elevated HDL-C levels compared with those in the MC group. Among all the PSP treatment groups, the 0.2 g/kg/d of PSP intervention (PSP-L group) had the greatest therapeutic effect on lowering the serum TG concentration, and the 0.4 g/kg/d of PSP intervention (PSP-M group) had the greatest effect on lowering the LDL-C concentration. However, there was no significant difference in improving HDL-C and TC levels among the different PSP intervention groups. These findings showed that PSP dose-independently improved the blood lipid levels in mice with T2DM induced by STZ.

### 3.4. PSP Effects on Pro-Inflammatory Cytokine and the Histopathologic Change in the Liver and Pancreas

**Figure 7:**
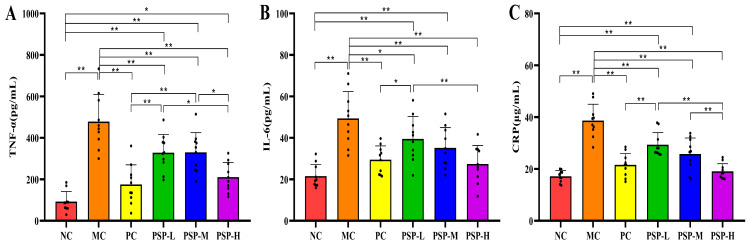
PSP effects on the levels of the pro-inflammatory cytokines (**A**) TNF-α, (**B**) IL-6, and (**C**) CRP. Note: * *p* < 0.05 or ** *p* < 0.01 indicates significant differences between groups, black dot (·) represents a data point.

As shown in Figure 7A–C, T2DM mice presented an enhanced inflammatory response with elevated serum IL-6, TNF-α, and CRP levels. At the experimental endpoint, these pro-inflammatory cytokine levels in all the drug treatment groups of mice decreased compared to the MC group (*p* < 0.05). Furthermore, the IL-6 and CRP contents in the three PSP treatment groups varied inversely with the PSP dose from low to high, and no significant difference in the IL-6 and CRP levels was found between the PSP-H group and the NC group.

**Figure 8:**
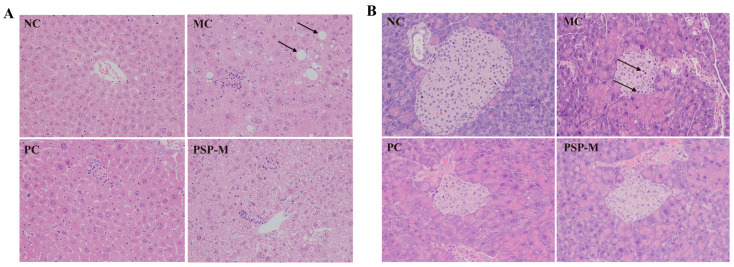
The histopathology of the liver (**A**) and pancreas (**B**) (400× magnification). Note: arrow (**A**) indicates macrovesicular vacuolation of hepatocytes, arrow (**B**) indicates islet cell necrosis and mononuclear cell infiltration.

Compared with the NC group, the histopathological analysis of the MC group showed hepatocytes with macrovesicular and microvesicular vacuolation changes. The scattered small foci of hepatocellular necrosis surrounded by inflammatory cell infiltration were also noted, and the infiltrates were composed mainly of lymphocytes with fewer neutrophils or eosinophils (Figure 8A). After drug treatment, liver tissues in the PC and PSP-M groups presented a relatively normal morphology of the liver and a reduced number of hepatocytes with macrovesicular and microvesicular vacuolation. In addition, in the MC group, the histopathological analysis of the pancreas revealed significant islet atrophy, as represented by reduced islet sizes and irregular shapes. The islet cells also decreased in number. Islet cell necrosis and mononuclear cell infiltration were noted (Figure 8B). Metformin and medium-dose PSP treatments attenuated pancreatic tissue damage in T2DM mice, and the PC and PSP-M groups presented a partial restoration of the islet structure without significant mononuclear cell infiltration.

### 3.5. PSP Effects on the Fecal SCFA Contents

Table 1 showed that the contents of fecal SCFAs from the MC group were significantly decreased compared to the NC group. After drug treatment, the contents of butyric acid and propionic acid of fecal samples from the mice in the PC and the three PSP groups, as well as the contents of acetic acid in the PC, PSP-M, and PSP-H groups, were higher than those in the MC group (*p* < 0.05), whereas the contents of isobutyric acid were not different between these five groups (*p* > 0.05).

### 3.6. PSP Effects on the Gut Microbiota

**Figure 9:**
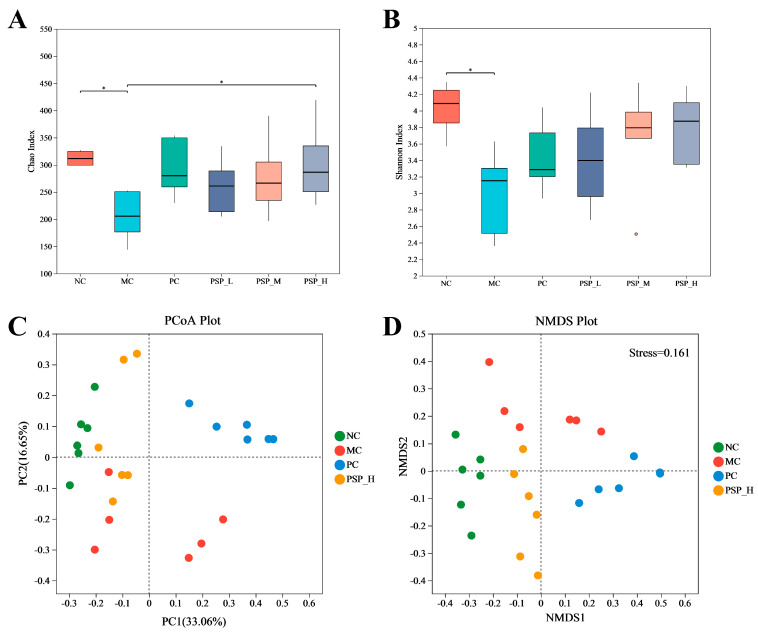
PSP effects on the gut microbiota diversity in T2DM mice. The α diversity is presented as the (**A**) Chao index and (**B**) Shannon index (* *p* < 0.05); the β diversity is presented as (**C**) PCoA and (**D**) NMDS (when stress is less than 0.2, NMDS accurately reflects the distribution of the data).

The Chao and Shannon indices of T2DM mice (MC group) were decreased compared with healthy mice (NC group) (*p* < 0.05) by the alpha diversity analysis, with the Chao index reflecting richness and the Shannon index reflecting the diversity of bacterial communities (Figure 9A,B). In the drug-treated groups of mice, these two indices increased to varying degrees, and the Chao index in the PSP-H group was remarkably increased compared with the MC group, indicating that PSP intervention in diabetic mice obviously improved the intestinal flora diversity. By examining the differences in the microbial community distribution in the fecal samples using nonmetric multidimensional scaling (NMDS) and principal coordinate analysis (PCoA), we found that the gut microbiota structure was altered in T2DM mice, as the MC and NC groups presented different clusters of samples (Figure 9C,D). After drug treatment, the clusters in the PC and PSP-H groups were distinctly separated from the cluster in the MC group, and the cluster in the PSP-H group was closer to that in the NC group, indicating that high-dose PSP-treated mice had a more similar gut microbiota structure compared to healthy mice.

**Figure 10:**
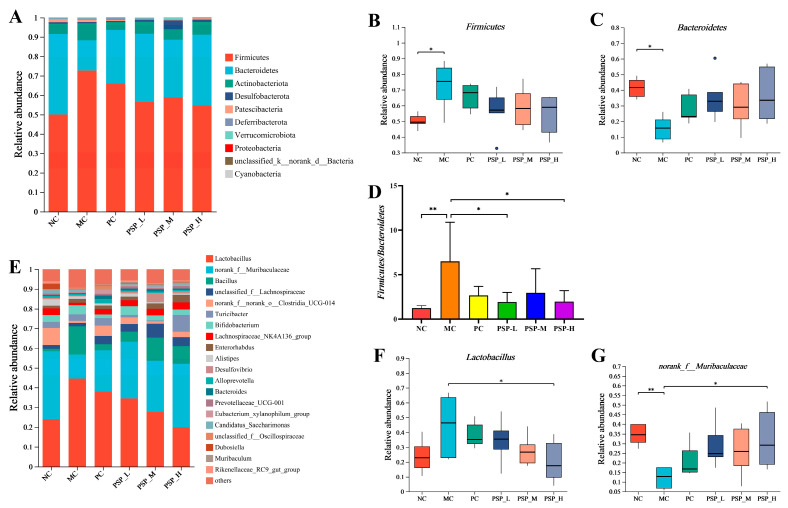
PSP effects on the components of the gut microbiota in T2DM mice. (**A**) Relative abundance of the gut microbiota at the phylum level; (**B**) differences in *Firmicutes* among the groups; (**C**) differences in *Bacteroidetes* among the groups; (**D**) ratio of *Firmicutes* to *Bacteroidetes*; (**E**) relative abundance of the gut microbiota at the genus level; (**F**) differences in *Lactobacillus* among the groups; (**G**) differences in *norank_f_Muribaculaceae* among the groups. * *p* < 0.05 and ** *p* < 0.01.

A total of 10 microorganisms (mainly *Firmicutes* and *Bacteroidetes*) with the highest abundance were detected in the fecal samples of each group at the phylum level (Figure 10A). The abundance of *Firmicutes* was higher and that of *Bacteroidetes* was lower in the MC group compared to the NC group (*p* < 0.05), whereas PSP and metformin treatment diminished this trend (Figure 10B,C). An increased ratio of *Firmicutes* to *Bacteroidetes* (F/B) has been shown to be more likely to lead to obesity [24]. Compared with that of healthy mice (NC group), the mean F/B value of T2DM mice (MC group) significantly rose. After medication, the mean F/B values decreased by 58.9%, 69.9%, 54.5%, and 70.0% in the PC, PSP-H, PSP-M, and PSP-L groups, respectively, compared with the MC group (Figure 10D). In addition, *Lactobacillus* and *norank_f_Muribaculaceae* were the dominant bacteria among the top 20 microorganisms at the genus level in terms of abundance in mouse fecal samples (Figure 10E). Figure 10F showed that the enrichment of *Lactobacillus* in T2DM mice was suppressed by interventions with PSP and metformin, with the high dose of PSP (PSP-H group) appearing to be more effective. As shown in Figure 10G, there was less *norank_f_Muribaculaceae* detected in the MC group compared with the NC group (*p* < 0.01). The relative abundance of this bacterium increased with drug treatment, and its abundance obviously differed between the PSP-H group and the MC group (*p* < 0.05). Therefore, our findings suggested that PSP could effectively modulate the gut microbiota structure in diabetic mice.

**Figure 11:**
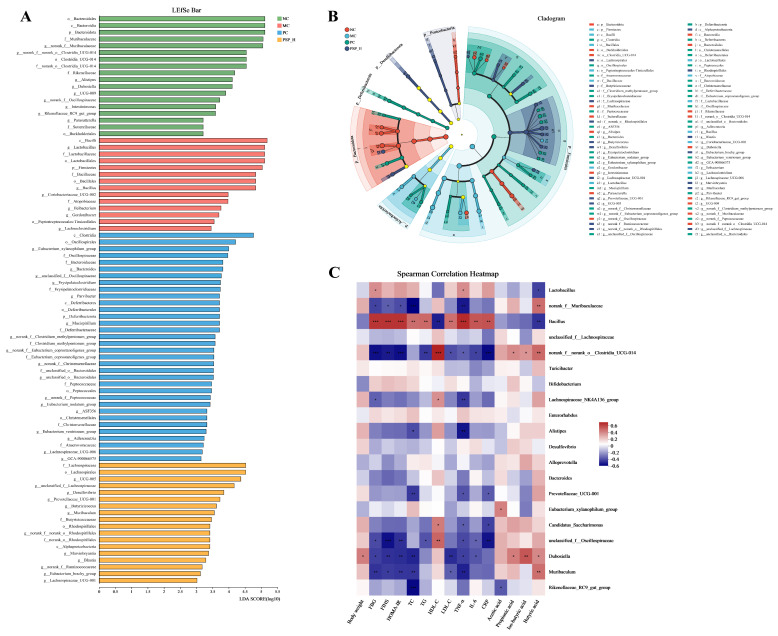
LEfSe analysis and correlation analysis. The LEfSe analysis results are presented as (**A**) an LDA discriminant bar graph and (**B**) an evolutionary branching graph (Different colors indicate different groups. Nodes of the same color represent components of the microbiota that play an important role and have the highest abundance in the group, whereas light yellow indicates no significant difference.). (**C**) Correlations between the gut microbiota (the top 20 microorganisms in terms of abundance at the genus level) and metabolic indicators in T2DM mice (red and blue nodes representing positive and negative correlations). * *p* < 0.05, ** *p* < 0.01, and *** *p* < 0.001.

LDA effect size (LEfSe) analysis (LDA score > 3, Figure 11A,B) revealed that the characteristic microbiota of the NC group were *norank__f__norank__o___Clostridia_UCG-014*, *norank__f__Muribaculaceae*, *Alistipes*, *Dubosiella*, *UCG-009*, *norank_f__Oscillospiraceae*, *Intestinimonas*, *Rikenellaceae_RC9_gut_group*, and *Parasutterella*. *Lactobacillus*, *Bacillus*, *Coriobacteriaceae_ UCG-002*, *Ileibacterium*, *Gordonibacter*, and *Lachnoclostridium* were the dominant microbiota in the MC group at the genus level. The most abundant microbiota in the PSP-H group at the genus level were *UCG-005*, *Eubacterium_brachy_group*, *unclassified_f_Lachnospiraceae*, *Desulfovibrio*, *Prevotellaceae_UCG-001*, *norank_f_Ruminococcaceae*, *Lachnospiraceae_UCG-001*, *Butyricicoccus*, *Muribaculum*, *norank_f_norank_o_Rhodospirillales*, *Marvinbryantia*, and *Blautia*. Furthermore, as shown in Figure 11C, the abundance of *Lactobacillus*, the dominant bacterium in the MC group, was positively associated with FBG and TNF-α levels and negatively associated with the SCFA content, especially the butyric acid content (*p* < 0.05) by Spearman’s correlation analysis. Similarly, *Bacillus* was strongly positively correlated with disorders of glycolipid metabolism and inflammatory response-related indicators but strongly negatively associated with butyric acid levels (*p* < 0.01). In contrast, *Muribaculum*, the predominant microorganism in the PSP-H group, was significantly negatively associated with the levels of FBG, FINS, HOMA-IR, LDL-C, TC, and TNF-α and positively correlated with butyric acid levels. Moreover, another characteristic bacterium in this group, *Prevotellaceae_UCG-001*, was significantly negatively associated with the levels of TC, TNF-α, and CRP.

## 4. Discussion

Numerous studies have confirmed that TCM could effectively prevent and manage T2DM [8]. PSP, a bioactive macromolecule from Chinese medicinal herbs, is becoming increasingly attractive because of its enormous potential applications as a functional food and drug with various health-promoting functions. PSP is beneficial in HFD-induced obese rats, as it improves hyperlipidemia, decreases BW, hepatic malondialdehyde, and inflammatory cytokine levels, and increases the hepatic catalase and superoxide dismutase activities [15]. PSP also ameliorates nonalcoholic fatty liver disease in rats [15,25]. Metformin is considered the first-line pharmacological treatment for T2DM in most guidelines and is used daily by more than 200 million patients [26]. In this experiment, treatment effects with PSP on T2DM were evaluated by comparing it with metformin, which was used as a positive control. The findings showed that PSP effectively attenuated weight loss in T2DM mice and decreased average food and water intake, consistent with the results of Wang et al., indicating that clinical symptoms such as polydipsia, polyphagia, polyuria, and weight loss were ameliorated in diabetic rats treated with PSP after 12 weeks [27]. In addition, we observed that after 4 weeks of PSP treatment, FBG levels were markedly lower in T2DM model mice with the improved OGTT results, indicating that PSP had the pharmacological ability to restore the disordered state of glucose metabolism, similar to the effects of other polysaccharides, such as *Hericium erinaceus* polysaccharides and *Holothuria leucospilota* polysaccharides, on diabetic animals [28,29].

As the main pathogenesis of T2DM, IR generally manifests as the hyporesponsiveness of body tissues to insulin action [30]. During the present study, three doses of PSP markedly reduced the FINS and HOMA-IR values, and the same therapeutic effect was achieved with moderate doses of PSP and metformin. These results suggest that PSP has therapeutic potential for improving insulin resistance in T2DM mice. As a disease closely linked with disorders of lipid metabolism, the symptoms of T2DM include reduced HDL-C levels and increased LDL-C, TC, and TG levels [31]. Our results revealed that PSP treatment obviously decreased the LDL-C, TC, and TG levels and increased the HDL-C level, suggesting that PSP affects lipid metabolism. Zeng et al. reported that PSP (200, 400, and 800 mg/kg) dose-dependently improved the blood lipid levels in HFD-induced obese rats [15]. Inconsistent with their findings, our research revealed that the different doses of PSP induced non-significant affection in improving serum TC and HDL-C levels in T2DM mice, and low-dose PSP was more effective than high-dose PSP in lowering TG levels, indicating that PSP effects on lipid levels in T2DM mice were dose-independent.

The results from a previous report indicated that the levels of inflammatory factors in T2DM patients were significantly increased and affected the sensitivity of insulin target tissues through a variety of mechanisms [32]. At the end of our study, the levels of inflammatory indicators (CRP, TNF-α, and IL-6) in the serum were markedly lower postdosing, which supported the hypothesis that PSP intake helps attenuate the body’s inflammatory response to alleviate the development of T2DM. Moreover, the administration of PSP mitigated organ damage in T2DM mice, including attenuating hepatic steatosis, restoring the pancreatic islet structure, and reducing inflammatory cell infiltration. Given that hepatic steatosis is a risk factor leading to further liver diseases, our findings suggest that PSP intake may be a useful strategy to protect against liver damage in diabetic patients [33].

Natural active polysaccharides are difficult to digest and absorb directly by the body, usually decomposed by the gut microbiota into SCFAs (acetic, propionic, and butyric acids, etc.) before entering the circulation [19]. Accumulating evidence suggests that SCFAs intervene in the development of T2DM in the stimulation of the peptide YY (PYY) and glucagon-like peptide-1 (GLP-1) secretion by colonocytes, which helps regulate host satiety and insulin secretion [34,35]. Moreover, SCFAs can increase intestinal gluconeogenesis with butyric acid and propionic acid facilitating the intestinal gluconeogenesis-related gene expression [36]. Furthermore, butyric acid is a major source of energy for colonocytes and induces the expression of intestinal endothelial tight junction proteins in maintaining the intestinal barrier integrity [37,38]. Unfortunately, SCFAs are commonly underproduced in T2DM patients. Notably, Hu et al. reported that the intake of antidiabetic drugs such as metformin and berberine promoted the production of SCFAs in obese mice [39]. Similar results were obtained in our study, where PSP treatment reversed the trend of decreased SCFA production in diabetic mice, significantly increasing the acetic, butyric, and propionic acid levels. Our findings showed that increasing the levels of SCFAs might be an important strategy to ameliorate metabolic diseases.

Previous studies have shown that the gut microbiota are an important risk factor for diabetes mellitus progression and severity [5,40]. Another report from Młynarska et al. indicates that gut microbial imbalances are involved in the pathogenesis of T2DM through multiple pathways, including effects on host glucolipid metabolism, inflammatory responses, insulin sensitivity, and intestinal permeability, suggesting that restoring the host–gut microbial relationship is highly important for treating diabetes mellitus [41]. In this study, PSP treatment markedly affected the intestinal flora diversity and altered the microbiota structure in T2DM mice, as determined via the alpha diversity analysis, PCoA, and NMDS analysis. Dominant organisms in the human gastrointestinal tract include *Firmicutes* and *Bacteroidetes*, constituting the majority of the total microbiota at the phylum level [42]. Moreover, the F/B ratio is used for evaluating intestinal homeostasis as a critical indicator, and decreasing this ratio helps improve intestinal permeability, inflammation, and endotoxemia [43]. Our results revealed that the PSP intervention could increase the abundance of *Bacteroidetes* and reduce the relative abundance of *Firmicutes* in T2DM mice, together with the significantly decreasing F/B value, consistent with the findings of another study in which mulberry fruit polysaccharides alleviated hyperglycemia by reducing the increase in the F/B value in diabetic db/db mice [44]. The abundance of *Lactobacillus*, a dominant bacterium in diabetic mice, decreased in a dose-dependent way after the PSP intervention. Karlsson et al. also reported that *Lactobacillus* was significantly enriched in T2DM patients’ guts and was positively associated with blood glucose levels [45]. However, *Lactobacillus* is mostly regarded as a probiotic and plays the most important role in maintaining the intestinal biological barrier [46]. We hypothesize that *Lactobacillus* enrichment in the intestines of diabetic patients may be involved in mediating metabolic disturbances in the progression of diabetes, as *Lactobacillus* has recently been reported to induce or maintain mild inflammation [47]. Moreover, we found that *norank_f_Muribaculaceae*, a bacterium strongly negatively correlated with all obesity-related factors and functionally diverse in terms of carbohydrate degradation, appeared to be differentially elevated in T2DM mice after PSP treatment, with the best therapeutic effect being observed with a high dose of PSP [48,49].

Our LEfSe analysis results indicate that the characteristic flora of T2DM mice changed, with an increased abundance of conditionally pathogenic bacteria, such as *Coriobacteriaceae_UCG-002* and *Lachnoclostridium*. *Coribacteriaceae UCG-002* was shown to produce phenol and p-cresol, both of which are cytotoxic and disrupt the intestinal barrier [50]. In addition, *Lachnoclostridium* was positively associated with the levels of T2DM-related circulating metabolites, such as *N*-acetylglucosamine and hydroxyasparagine, which might exacerbate insulin resistance [51]. We also detected more *Bacillus* in the MC group and revealed that *Bacillus* was strongly positively correlated with disorders of glycolipid metabolism and inflammation-related indicators. However, the role that *Bacillus* plays in exacerbating the risk of diabetes mellitus remains to be confirmed. Notably, the structure of gut microbiota in T2DM mice was obviously altered by the high-dose PSP treatment, which enriched beneficial flora such as *Blautia* and *Muribaculum*. *Blautia* is reported to be a potential probiotic that promotes biological transformation and has positive effects on modulating host health and alleviating metabolic syndrome [52]. Moreover, *Muribaculum* is associated with BW regulation, and its abundance was shown to be negatively correlated with intestinal inflammation [53]. Interestingly, we found that most of the dominant microbiota in PSP-treated mice were related to SCFA production, such as *Lachnospiraceae* and *Butyricicoccaceae* at the family level, and *unclassified_f__Lachnospiraceae*, *Prevotellaceae_UCG-001*, *Butyricicoccus*, *Marvinbryantia*, *norank_f__Ruminococcaceae*, and *Lachnospiraceae_UCG-001* at the genus level. *Lachnospiraceae* contains *unclassified_f__Lachnospiraceae* and *Lachnospiraceae_UCG-001*, which are obligate anaerobic bacteria that affect the host by producing large amounts of butyric acid in the animal gut; these bacteria are involved in bile acid metabolism and improve the resistance to intestinal pathogen colonization [54]. In addition, the bacteria *Butyricicoccus* (belonging to the family *Butyricicoccaceae*) and *Marvinbryantia* are both important butyric acid producers, and an increase in *Marvinbryantia* abundance was consistent with a decrease in IR levels [55,56]. Moreover, *Prevotellaceae_UCG-001* and *norank_f__Ruminococcaceae* have also been shown to be related to SCFA production [57,58]. He et al. noted that increasing the abundance of SCFA-producing bacteria plays a crucial role in attenuating intestinal microbial disorders in diabetic mice [58].

Currently, most studies have focused on various bioactivities of PSP, including antiaging, antidepressant, anti-obesity, antidiabetic, antioxidant, and immunomodulatory activities [59]. However, knowledge of the molecular mechanism by which PSP protects against T2DM is limited and requires further exploration. Our next research focus is to elucidate the mechanism of action of PSP in preventing and treating STZ-induced T2DM in mice via metabolomic analysis and network pharmacology methods for scientific PSP utilization. Although the PSP effects on the gut microbiota in T2DM mice were evaluated in our research, we should further investigate whether the protective effects of PSP are mediated by intestinal microorganisms through fecal microbiota transplantation experiments.

## 5. Conclusions

Our results indicate that PSP could provide effective protection against T2DM by modulating glycolipid metabolism disorders and gut microbiota dysbiosis in an animal model. These changes include stabilizing BW, lowering FBG levels, improving glucose tolerance, hyperlipidemia, and insulin resistance, attenuating inflammatory responses, restoring the structures of the liver and pancreas, and increasing the levels of SCFAs. Furthermore, the PSP intervention could effectively improve the gut microbial diversity and regulate their composition in T2DM mice and increase the abundance of beneficial bacteria, especially SCFA-producing bacteria. These findings provide new perspectives on PSP as a potential adjuvant therapy for T2DM. However, the specific therapeutic mechanism and effect of a combined treatment with PSP and metformin have not yet been elucidated. Hence, additional preclinical studies are still necessary in the future to reduce the required dose of diabetes medications and improve their efficacy.

## Figures and Tables

**Table 1:** PSP effects on fecal SCFA profiles in T2DM mice.

Group	SCFA Production (mM)			
	Acetic Acid	Butyric Acid	Isobutyric Acid	Propionic Acid
NC	3.28 ± 1.08	0.49 ± 0.13	0.09 ± 0.03	0.38 ± 0.08
MC	1.92 ± 0.62 *	0.12 ± 0.08 **	0.04 ± 0.02 **	0.07 ± 0.04 **
PC	3.66 ± 0.93 ^##^	0.40 ± 0.14 ^##^	0.04 ± 0.02 **	0.24 ± 0.10 *^##^
PSP-L	2.80 ± 1.05	0.33 ± 0.12 ^#^	0.03 ± 0.02 **	0.19 ± 0.04 **^#^
PSP-M	3.07 ± 0.81 ^#^	0.29 ± 0.17 *^#^	0.05 ± 0.03 **	0.27 ± 0.13 ^##^
PSP-H	3.36 ± 0.99 ^#^	0.43 ± 0.17 ^##^	0.02 ± 0.01 **	0.26 ± 0.11 *^##^

Note: * *p* < 0.05 or ** *p* < 0.01 indicates significant differences between NC and MC, PC, PSP-L, PSP-M, and PSP-H; ^#^ *p* < 0.05 or ^##^ *p* < 0.01 indicates significant differences between MC and PC, PSP-L, PSP-M, and PSP-H.

## Data Availability

All relevant data are within this paper.
